# Rhizospheric Bacteria of Cover Legumes from Acidic Soils Are Capable of Solubilizing Different Inorganic Phosphates

**DOI:** 10.3390/microorganisms12061101

**Published:** 2024-05-29

**Authors:** Winston F. Ríos-Ruiz, Roy D. Casique-Huamanguli, Renzo A. Valdez-Nuñez, Jose C. Rojas-García, Anderson R. Calixto-García, Franz Ríos-Reátegui, Danny F. Pompa-Vásquez, Euler Padilla-Santa-Cruz

**Affiliations:** 1 Laboratorio de Microbiología Agrícola “Raúl Ríos Reátegui”, Departamento Académico Agrosilvopastoril, Facultad de Ciencias Agrarias, Universidad Nacional de San Martín, Tarapoto 22202, Peru; roydan.ch1993@gmail.com (R.D.C.-H.); jogarcia@unsm.edu.pe (J.C.R.-G.); df.pompava@unsm.edu.pe (D.F.P.-V.); ce.padilla@unsm.edu.pe (E.P.-S.-C.); 2 Laboratorio de Investigación en Biotecnología, Departamento Académico de Ciencias Básicas y Afines, Facultad de Ingeniería, Universidad Nacional de Barranca, Barranca 15169, Peru; rvaldez@unab.edu.pe (R.A.V.-N.); acalixtog182@unab.edu.pe (A.R.C.-G.); 3 Departamento Académico de Ingeniería Electrónica, Facultad de Ingeniería Electrónica y Eléctrica, Universidad Nacional Mayor de San Marcos, Lima 15081, Peru; franz.rios@unmsm.edu.pe

**Keywords:** acidic soils, phosphate-solubilizing bacteria, cover crop legumes, rhizosphere, plant-microorganism interactions, sustainability

## Abstract

Due to its adsorption with aluminum and iron hydroxides, phosphorus viability is low in acidic soils; thus, the aim of this study was to isolate and identify bacteria from the rhizosphere of four legumes growing in acidic soils of the Cumbaza Sub-basin, San Martín, Peru, as well as to characterize their ability to solubilize aluminum phosphate and iron phosphate. The isolation process was conducted on TSA medium and the isolates were classified based on their origin and morphocolonial characteristics, with the bacillary shape being the most frequent, followed by cocci. To assess the solubilization of aluminum and iron phosphates, the liquid medium GELP was employed. Sixteen strains were selected, among which three stood out for their effectiveness in solubilizing AlPO_4_ (Sfcv-098-02, 22.65 mg L^−1^; Sfc-093-04, 26.50 mg L^−1^; and Sfcv-041-01-2, 55.98 mg L^−1^) and one for its ability to solubilize FePO_4_ (Sfcr-043-02, 32.61 mg L^−1^). These four strains were molecularly characterized, being identified as *Enterobacter* sp., *Pseudomonas* sp., and *Staphylococcus* sp. Additionally, a decrease in pH was observed in the reactions, with values ranging from 5.23 to 3.29, which enhanced the phosphate of solubilization. This suggests that the selected bacteria could be used to improve phosphorus availability in agricultural soils.

## 1. Introduction

Rhizospheric bacteria play a fundamental role in plant health and productivity through establishing close symbiotic relationships with plant roots, specifically in the zone known as the rhizosphere. This beneficial relationship influences multiple aspects of plant growth, such as nitrogen fixation, nutrient solubilization, the production of indole acetic acid and siderophores, and protection against pathogens [1,2]. Additionally, cover crop legumes represent a valuable tool for promoting sustainable agricultural practices, providing multiple benefits for soil health, biodiversity, and agricultural productivity [3]. Their capacity to conserve nutrients, improve soil quality, and enhance the efficiency of agricultural systems makes them a key component in transitioning towards more sustainable and resilient agriculture [4].

Plants face several challenges in acidic soils, primarily related to the availability of essential nutrients. One of the main issues is the soil’s reduced capacity to retain and supply important nutrients for plant growth, such as phosphorus [5]. This element is one of the most-required nutrients by plants (second only to nitrogen), being a fundamental component of nucleic acids (RNA and DNA), phospholipids (forming cell membranes), and adenosine triphosphate (ATP), as well as playing a key role in processes such as respiration and photosynthesis [6]. An effective strategy for improving soil fertility involves the utilization of microorganisms, including bacteria, which are capable of decomposing insoluble forms of phosphorus (e.g., the phosphates present in the soil), thus converting them into soluble forms that plants can easily absorb [7].

In acidic conditions, phosphorus tends to precipitate and become fixed in insoluble forms, such as aluminum (AlPO_4_) and iron (FePO_4_) phosphates, which are not usable by plants [8]. As a result, plants may experience phosphorus deficiencies, which negatively impact their growth, development, and yield. Furthermore, soil acidity can influence the activity of the rhizosphere bacterial community and the availability of other nutrients, further exacerbating nutritional problems in plants [9]. Improving the availability of phosphorus and other nutrients in acidic soils is important to ensure healthy plant growth and maximize agricultural productivity under these adverse conditions [10]. Given the importance of phosphorus in agriculture and soil recovery processes, the search for sustainable alternatives to phosphate fertilizers is essential to ensure adequate crop productivity [11].

Rhizospheric bacteria play a crucial role in the solubilization of inorganic phosphates, thus enhancing the availability of phosphorus for host plants (especially in acidic soils), as they possess enzymes (e.g., phosphatases) which catalyze the release of phosphate from insoluble forms present in the soil [12,13]. Furthermore, some rhizospheric bacteria, including those from the genera *Pseudomonas* sp., *Bacillus* sp., *Brevibacillus* sp., *Paenibacillus* sp., and *Brevibacterium* sp. [14], have the ability to secrete organic acids such as lactic acid, acetic acid, succinic acid, and palmitic acid, which can acidify the surrounding environment, thus solubilizing aluminum and iron phosphates which are typically more insoluble in acidic soils [15]. Likewise, through increasing the accessibility of phosphorus for plants, these bacteria can reduce the need for phosphate fertilizers. This approach not only generates economic benefits for farmers but also has a positive environmental impact through reducing the pollution associated with chemical fertilizers [16]. The San Martín region in Peru encompasses 534,383 hectares dedicated to agricultural crops [17], and many of these areas have acidic soils, such as those in the Cumbaza sub-basin [18] (from which the strains of phosphate-solubilizing bacteria evaluated in this study originated). Therefore, there is a need to conduct studies to understand the presence of plant growth-promoting microorganisms that enable the implementation of strategies contributing to sustainable agriculture.

The aim of the present study was to isolate, characterize, and evaluate the phosphate solubilization capacity of rhizospheric bacteria from four cover crop legumes—*Cajanus cajan*, *Canavalia ensiformis*, *Crotalaria juncea*, and *Vigna unguiculata*—growing in six degraded soils from the Cumbaza sub-basin, San Martin. The test was carried out using two insoluble phosphate sources (aluminum phosphate and iron phosphate). The hypothesis of this investigation was that phosphate-solubilizing bacteria exist in the soils of the Cumbaza sub-basin, which are capable of associating with the rhizosphere of cover crop legumes and could potentially allow for the future production of bioinoculants using these bacteria.

## 2. Materials and Methods

### 2.1. Study Area and Soil Sampling

**Figure 1:**
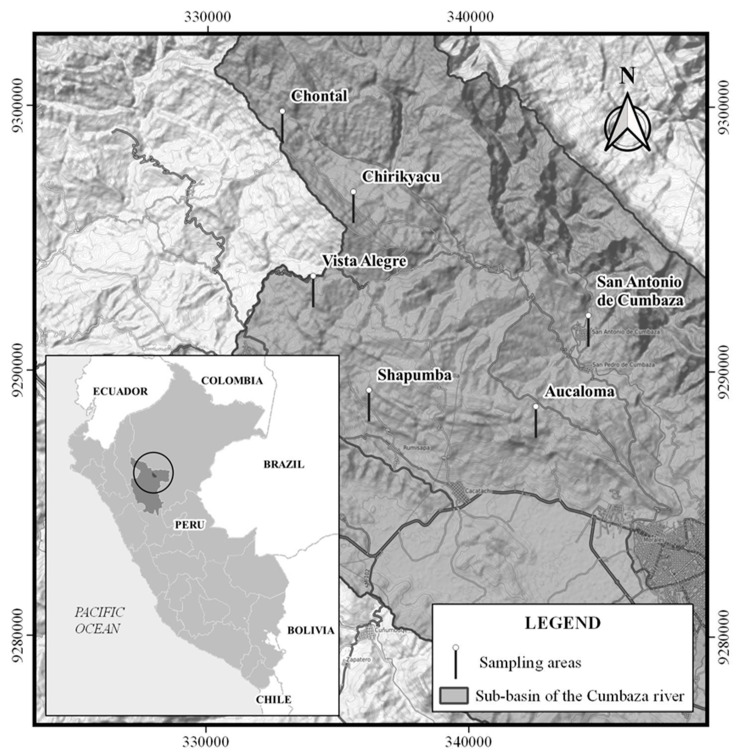
Map of the Cumbaza River sub-basin in San Martín, Peru, showing the location of the 6 soil sampling zones (Extracted and adapted from [18,19]). The circle in the smaller box represents the area of San Martín where the Cumbaza sub-basin is located.

Soil samples were collected from six zones in the Cumbaza sub-basin: Aucaloma, Chirikyacu, Chontal, San Antonio de Cumbaza, Shapumba, and Vista Alegre (Figure 1). According to [18], these soils are acidic and of low fertility. One kilogram of soil was extracted from 10 equidistant points in each zone, totaling 30 sub-samples per zone, at a depth of 0–20 cm. A composite sample was formed from these sub-samples, which was subsequently homogenized. Two 1 kg sample fractions were taken for microbiological and physicochemical analyses. Additionally, 20 kg of soil was collected at each sampling point for the cultivation of cover crop legumes under greenhouse conditions.

### 2.2. Greenhouse Trial

The soils collected from the six study zones were air-dried at ambient temperature (between 20 and 25 °C) in the shade, then they were crushed and sieved through a fine mesh with 2 mm openings. To prevent compaction in the pots, they were mixed with sterilized vermiculite in a 1:1 *v*/*v* ratio and placed in 3.5 kg capacity pots. Four cover crop legumes adapted to tropical climates—namely, *C. Cajan*, *C*. *ensiformis*, *C*. *juncea*, and *V*. *unguiculata*—were sown after disinfection according to [20]. The plants were kept in a greenhouse for 45 days for *V*. *unguiculata* and 60 days for *C*. *Cajan*, *C*. *ensiformis*, and *C. juncea*, being watered with distilled water until field capacity was reached. After this period, rhizospheric and root soil samples were collected, which were placed in single-use bags to isolate rhizospheric bacteria. 

### 2.3. Isolation of Bacteria from Rhizospheric Soils

For the isolation of rhizospheric bacteria, rhizospheric soil samples obtained from *V*. *unguiculata*, *C*. *Cajan*, *C*. *ensiformis*, and *C. juncea* were processed, according to [21], by means of surface plating on Tryptone Soy Agar (TSA) medium. This medium consisted of pancreatic digest of casein (15 g), papaic digest of soy bean (5 g), sodium chloride (5 g), Agar-agar (15 g), and distilled water (1000 mL). Then, 10 g of rhizospheric soil was mixed with 90 mL of sterile physiological saline solution (SSFe). Subsequently, a series of dilutions were made, starting with a 1:100 dilution and continuing until a 1:1,000,000 dilution was reached. From the last three dilutions (10^−4^, 10^−5^, and 10^−6^), 0.1 mL was plated onto Petri dishes containing TSA medium. The Petri dishes were then incubated at room temperature for approximately 1 week. Subsequently, plates showing between 30 and 300 colony-forming units (CFUs) were considered for the selection of isolated colonies with different morphocolonial characteristics. These colonies were sub-cultured on TSA medium as needed, until pure colonies with similar morphologies were obtained. The isolated colonies underwent microscopic evaluations (Gram staining and bacterial shape) and macroscopic evaluations (colony diameter, color, surface, elevation, shape, margin, and consistency), following the protocol described by [22]. The strains were then preserved at −20 °C until later re-activation for future studies.

### 2.4. Evaluation of Phosphate Solubilization by Rhizospheric Bacteria

For the assessment of phosphate solubilization ability, re-activated strains were inoculated into TSB liquid medium (tryptone, 15 g; peptone, 5 g; NaCl, 5 g; distilled H_2_O, 1000 mL; pH, 7.0) and incubated at 30 °C for 24 h. Cellular suspensions were adjusted with the same sterile TSB liquid medium to reach an OD_600nm_ of 0.5, thus constituting the inoculum. The *Rhizobium tropici* CIAT 899 strain was used as a positive control for solubilization, following the protocol reported by [23]. The liquid medium used to assess the solubilization of aluminum phosphate and iron phosphate was the GELP medium [21] (glucose, 10 g; peptone, 5 g; yeast extract, 0.05 g), soil extract (100 mL; filtered supernatant of 1 kg soil in 1 L distilled water, autoclaved and allowed to stand 48 h), MgSO_4_ 10% (2 mL), CaCl_2_ 10% (2 mL), NaCl 10% (1 mL), micronutrient solution (2 mL) constituted by (Na_2_MoO_4_·2H_2_O, 0.200 g; MnSO_4_·H_2_O, 0.235 g; H_3_BO_3_, 0.280 g; CuSO_4_·5H_2_O, 0.008 g; ZnSO_4_·7H_2_O, 0.024 g dissolved in 200 mL distilled water), Fe-EDTA 1.64% (4 mL), yeast extract (0.05 g), and agar (15 g as solid medium), to which FePO_4_ (0.89 g) was added for iron phosphate solubilization and AlPO_4_ (0.89 g) for aluminum phosphate solubilization testing. The solutions without the strains were considered as the blank treatments. A total of 0.5 mL of the inoculum was inoculated into 50 mL of GELP medium per strain, with three repetitions, containing the corresponding insoluble phosphate source. The culture was incubated at 28 °C for 5 days with agitation at 130 rpm. At the end of the evaluation period (120 h), each sample was centrifuged at 13,000 rpm for 5 min, and the supernatants were stored for phosphate solubilization studies and pH measurements.

The determination of phosphate solubilization was carried out following the phosphomolybdate method described by [24]. Aliquots of 1200 µL were taken in triplicate from the growing culture medium, which were subsequently centrifuged at 13,000 rpm for 5 min in a centrifuge (Mikro200 Hettich, Tuttlingen, Germany), in order to obtain the supernatant containing soluble phosphate. In a cuvette, 120 µL of a reactive solution composed of 10 mL of 3% ammonium heptamolybdate, 25 mL of 13.5% concentrated sulfuric acid, 10 mL of 5% ascorbic acid, and 5 mL of 0.14% potassium antimony tartrate was mixed with 1000 µL of the centrifuged supernatant. This mixture was incubated at room temperature for 10 min. The formation of a blue color indicated the presence of phosphates, and this color remained stable for up to two hours. Absorbance was measured at 655 nm using a spectrophotometer (Thermofisher, Spectronic 200, Suwa, Japan). Absorbance values were correlated with a standard phosphate curve to determine the concentration (in µg mL^−1^ or mg L^−1^) of phosphates in the sample. After each sampling, the pH was measured in 2 mL of supernatant using a pH meter (WTW XYLEM, inoLab 7110, Oberbayern, Germany).

### 2.5. Molecular Characterization of Phosphate-Solubilizing Bacteria

The phosphate-solubilizing bacteria strains that excelled in phosphate solubilization—including Sfcv-041-01-2, sfcv-098-02, and sfcv-041-01-2 for AlPO_4_ solubilization and Sfcr-043-02 for FePO_4_ solubilization—underwent characterization through amplification and sequencing of the 16S rRNA gene. For this purpose, the isolated bacteria, preserved in glycerol at −20 °C, were re-activated in TSA medium and then cultivated in TSB medium at 30 °C and 120 rpm until reaching the logarithmic growth phase (OD_600nm_~0.6). Next, the cells were collected through centrifugation at 12,000 rpm for 3 min at room temperature. Genomic DNA extraction was performed using the GenElute Bacterial Genomic DNA kit from Sigma-Aldrich (St. Louis, MO, USA), following the manufacturer’s instructions, and quantification was carried out with a NanoDrop ND1000 spectrophotometer (Thermo Fisher Scientific, Waltham, MA, USA). Amplification of the 16S rRNA gene was carried out through PCR with the primers fD1 (CCGAATTCGTCGACAACAGAGTTTGATCCTGGCTCAG) and rD1 (CCCGGGATCCAAGCTTAAGGAGGTGATCCAGCC), under the conditions described by [25]. PCR products were visualized on 0.7% agarose gels prepared in TBE buffer solution and photographed under UV light after electrophoresis. The sequencing of the amplicons was carried out by Macrogen (Seoul, Republic of Korea). The obtained sequences were edited and compared with those deposited in GenBank using the National Center for Biotechnology Information (NCBI) BLAST (http://www.ncbi.nlm.nih.gov/genbank/, accessed on 10 February 2024). For taxonomic identification of 16S rRNA gene sequences of type species, EZ-Taxon [26] was used. Phylogenetic analysis was carried out with MEGA6 [27], using Clustal W for alignments, the [28] 2-parameter model for distance calculation, and the neighbor-joining algorithm for phylogenetic tree construction, with bootstrapping based on 1000 repetitions to estimate confidence. Similarity tables were constructed using Phydit version 3.1 [29], employing Clustal X to align the sequences.

### 2.6. Research Design and Statistical Analysis

To evaluate the solubilization efficiency, a completely randomized design was employed. The number of treatments was determined based on the number of isolated and purified bacteria, resulting in 18 treatments, including a positive control (*Rhizobium tropici* CIAT 899) and a negative or blank control (culture medium plus phosphate without strain). All bacterial strains were evaluated on two insoluble phosphate sources (AlPO_4_ and FePO_4_). All experiments were conducted in triplicate to assess reproducibility, and the results are expressed as mean values from the difference between the value of available phosphate in the inoculated treatment minus the concentration of available phosphate in the control treatment. The treatments that showed absorbance levels equal to or lower than the blank were considered as treatments without phosphate solubilization. The normality of the data was checked using the Shapiro–Wilks test, and standard deviations and experimental standard errors were calculated. The results underwent statistical analysis using analysis of variance, correlation coefficients (r), and tests were conducted using the Duncan test (*p* < 0.05) to determine significance.

## 3. Results

### 3.1. Isolation and Morphocolonial Characterization of Rhizospheric Bacteria

Four strains of *V. unguiculata*, eight strains of *C*. *ensiformis*, and four strains of *C*. *juncea* were selected and isolated (Table 1). These selected strains were deposited in the culture collection of the Agricultural Microbiology Laboratory “Raúl Ríos Reátegui” at the Faculty of Agricultural Sciences of the Universidad Nacional de San Martín, along with their respective accession numbers, making them available for future research.

**Figure 2:**
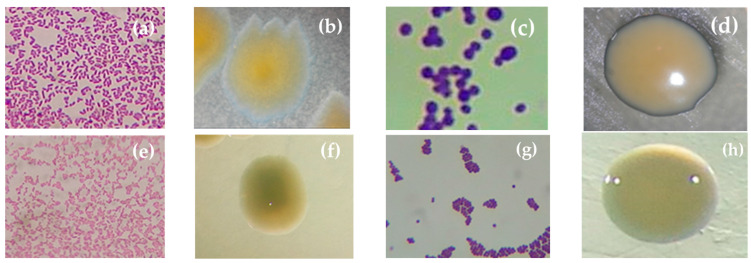
Representative samples of morphocolonial characterization of rhizospheric bacteria. (**a**) Gram-negative bacilli, 1000×, and (**b**) colony of strain Sfcv-041-01-2; (**c**) Gram-positive cocci, 1000×, and (**d**) colony of strain Sfc-093-04; (**e**) Gram-negative bacilli, 1000×, and (**f**) colony of strain Sfcr-043-02; (**g**) Gram-positive cocci, 1000×, and (**h**) colony of strain Sfcv-098-02.

Diverse colonial morphologies were observed among the 16 isolates, with the bacillary shape being the most frequent, followed by cocci. According to the results of the Gram staining, it was determined that bacteria with a bacillary shape were Gram-negative in 81.25%, while those with a coccus shape were Gram-positive in 18.75% (Table 2, Figure 2).

### 3.2. Solubilization of AlPO_4_ and FePO_4_ by Rhizospheric Bacteria

#### 3.2.1. Solubilization of AlPO_4_ by Rhizospheric Bacteria

Duncan’s mean comparison analysis for AlPO_4_ solubilization (in mg L^−1^) revealed significant differences among treatments. Strain Sfcv-041-01-2 stood out with a superior solubilization ability of 55.98 mg L^−1^, compared to the other strains (Table 3). This strain was followed by strains Sfc-093-04 and Sfcv-098-02, with 26.50 mg L^−1^ and 22.65 mg L^−1^, respectively, being statistically equivalent to each other. On the other hand, strains Sfcv-41-9 and Sfcv-129-01 exhibited the lowest AlPO_4_ solubilization ability, with 6.41 mg L^−1^ and 5.98 mg L^−1^, respectively. Table 3 indicates that the pH of the cultured supernatants ranged from 5.23 to 4.03 at the end of the experiment.

#### 3.2.2. Solubilization of FePO_4_ by Rhizospheric Bacteria

In the Duncan’s mean comparison analysis for FePO_4_ solubilization, significant differences were observed among treatments. Strain Sfc-159-01 stood out with a superior solubilization ability of 37.31 mg L^−1^, followed by strain Sfcv-041-9 with 34.96 mg L^−1^ (Table 3). On the other hand, strains Sfc-070-04 and Sfcv-041-01-2 showed the lowest values, with 17.22 mg L^−1^ and 15.36 mg L^−1^, respectively. Table 3 indicates that the pH ranged between 5.37 and 3.29 at the end of the experiment. All strains reduced the medium pH compared to that of the strain-free control (blank), which was 7.0.

### 3.3. Molecular Characterization of Phosphate-Solubilizing Bacteria

**Figure 3:**
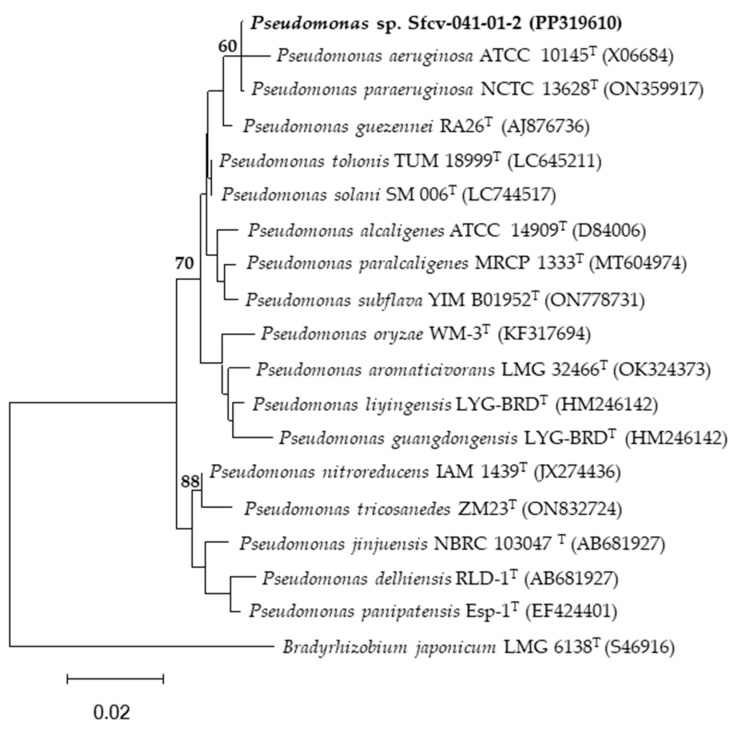
Maximum-likelihood phylogenetic tree based on 16S rDNA gene sequences (1556 positions) showing the relationships among *Pseudomonas* strain isolated from the San Martin region and closely related species of the genus *Pseudomonas*. The significance of each branch is indicated by a bootstrap value (as percentage) calculated for 1000 subsets (only values greater than 50% are indicated). The position of strain Sfcv-041-01-2 is highlighted in bold in the phylogenetic tree. Bar, 2 substitutions per 100 nucleotide positions. The 16S rDNA sequence of *Bradyrhizobium japonicum* LMG 6138^T^ was used as the outgroup.

**Figure 4:**
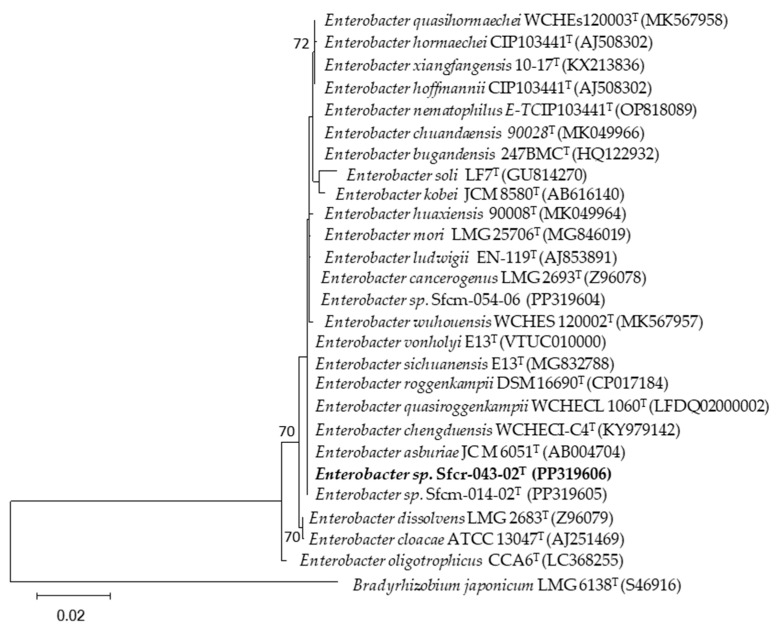
Maximum-likelihood phylogenetic tree based on 16S rDNA gene sequences (1556 positions) showing the relationships among *Enterobacter* strains isolated from the San Martin region and closely related species of the genus *Enterobacter*. The significance of each branch is indicated by a bootstrap value (as percentage) calculated for 1000 subsets (only values greater than 50% are indicated). The position of strain Sfcr-043-02^T^ is highlighted in bold in the phylogenetic tree. Bar, 2 substitutions per 100 nucleotide positions. The 16S rDNA sequence of *Bradyrhizobium japonicum* LMG 6138^T^ was used as the outgroup.

**Figure 5:**
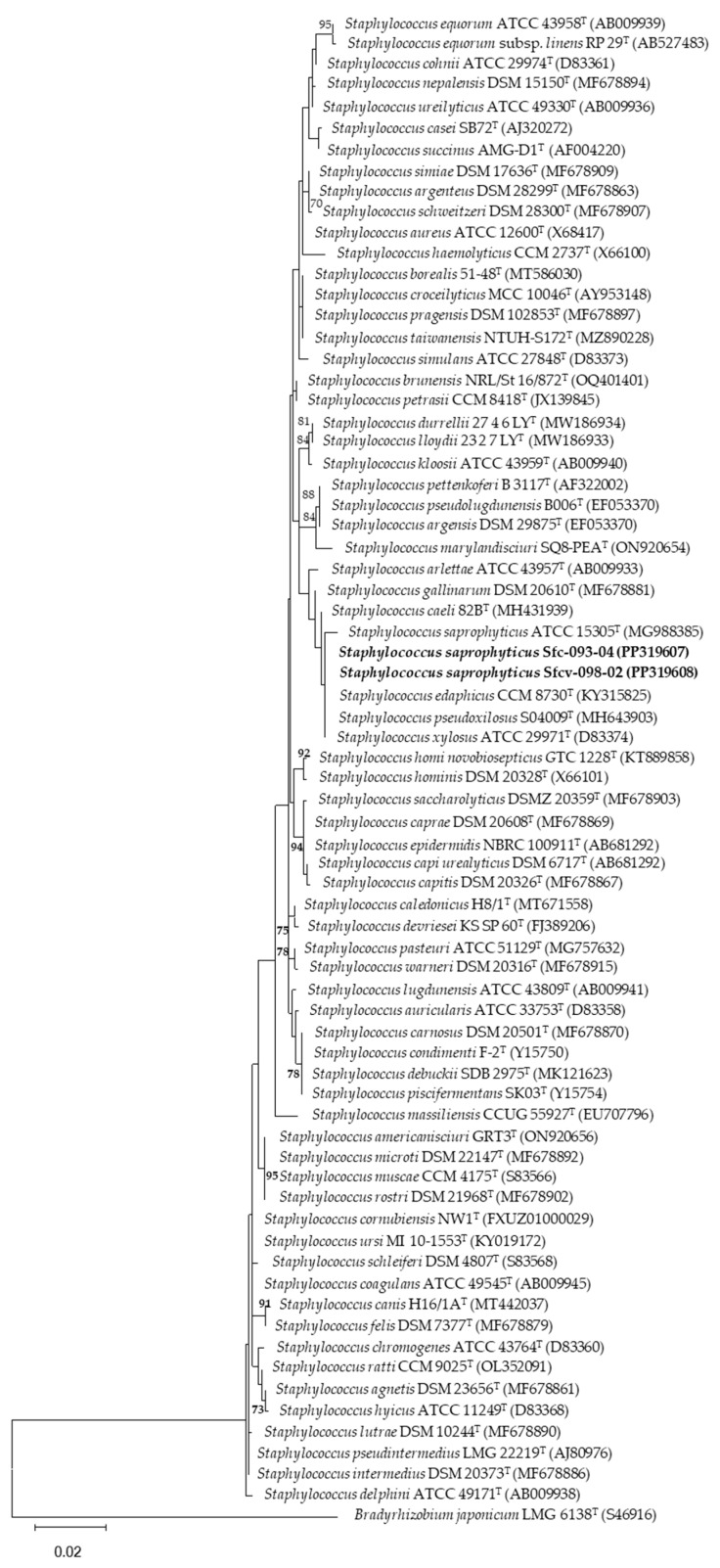
Maximum-likelihood phylogenetic tree based on 16S rDNA gene sequences (1556 positions) showing the relationships among *Staphylococcus* strains isolated from the San Martin region and closely related species of the genus *Staphylococcus*. The significance of each branch is indicated by a bootstrap value (as percentage) calculated for 1000 subsets (only values greater than 50% are indicated). The positions of the Sfc-093-04 and Sfcv-098-02 strains are highlighted in bold in the phylogenetic tree. Bar, 2 substitutions per 100 nucleotide positions. The 16S rDNA sequence of *Bradyrhizobium japonicum* LMG 6138^T^ was used as the outgroup.

Figure 3, Figure 4 and Figure 5 show the phylogenetic trees based on the partial sequence of the 16S ribosomal gene of the four bacterial strains highlighted for their phosphate solubilization ability, both with respect to aluminum (*Pseudomonas* sp. Sfcv-041-01-2, PP319610; *Staphylococcus saprophyticus* Sfc-093-04, PP319607; and *Staphylococcus saprophyticus* Sfcv-098-02, PP319608) and iron (*Enterobacter* sp. Sfcr-043-02, PP319606) phosphates.

The BLAST analysis revealed that the isolates belong to the genera *Enterobacter*, *Pseudomonas*, and *Staphylococcus* (Table 4). The isolate Sfcr-043-02 from the Chontal area showed a close relationship with *Enterobacter sichuanensis* WCHECL1597^T^, *Enterobacter roggenkampii* DSM 16690^T^, *Enterobacter quasiroggenkampii* WCHECL 1060^T^, and *Enterobacter vonholyi* E13^T^, with 99.86% identity. Similarly, isolate Sfcv-041-01-2, also from the Chontal area, exhibited high similarity with *Pseudomonas aeruginosa* JCM 5962^T^ and *Pseudonomas paraeruginosa* PA7^T^, with 99.93% identity. On the other hand, isolate Sfc-093-04 from the Aucaloma area showed a close relationship with *Staphylococcus saprophyticus* ATCC 15305^T^, with 100% identity, while isolate Sfcv-098-02 from the Shapumba area was also closely related to *Staphylococcus saprophyticus* ATCC 15305^T^, with 100% identity. The generated molecular datasets have been deposited in NCBI GenBank (http://www.ncbi.nlm.nih.gov/genbank/, accessed on 10 February 2024) with their corresponding accession numbers (Table 4). 

## 4. Discussion

### 4.1. Isolation and Morphocolonial Characterization of Rhizospheric Bacteria

Variations in the frequency of isolates of rhizospheric bacteria observed among different legumes are likely due to the unique root exudate profile of each plant. These profiles attract distinct microbial communities, turning the rhizosphere into an “exclusive” zone of feeding and interaction that influences plant growth [30]. This work constitutes the first report on the isolation of rhizospheric bacteria capable of solubilizing insoluble phosphates from the rhizosphere of cover legumes in degraded acidic soils of the tropical region of Peru. It is worth noting that the isolation of rhizospheric bacteria was also studied by the authors of [31], who found rhizospheric bacteria in solid phosphate sludges from the Khuribga phosphate mining center, Morocco, determining that all were Gram-negative. Similarly, the authors of [8] investigated the solubilization of calcium, aluminum, and iron phosphates by native bacteria of the genera *Bacillus* and *Burkholderia* in acidic soils of Odisha, India. These authors highlighted the importance of selecting beneficial microbes native to the area, which are adapted to the local climatic environment.

### 4.2. Solubilization of AlPO_4_ by Rhizospheric Bacteria

Previous studies on aluminum phosphate solubilization have reported values lower than those obtained in our research. For instance, the authors of [8] recorded a maximum solubilization of 58.49 mg L^−1^, while the authors of [31] achieved a maximum of 20.60 mg L^−1^. However, in our study, the strain *Pseudomonas* sp. Sfcv-041-01-2 (PP319610) demonstrated a solubilization capacity of 55.98 mg L^−1^, and the strains *Staphylococcus saprophyticus* Sfc-093-04 (PP319607) and Sfcv-098-02 (PP319608) registered solubilization levels of 26.50 mg L^−1^ and 2.65 mg L^−1^, respectively. These variations could be attributed to the specific soil and climatic conditions of the locations from which the native bacteria were isolated.

A positive correlation was observed between soluble phosphorus and the pH of the cultured supernatant. An example of this phenomenon is represented by the strains *Pseudomonas* sp. Sfcv-041-01-2 (PP319610) and *Staphylococcus saprophyticus* Sfc-093-04 (PP319607), which exhibited maximum solubilization and also presented the highest pH values, surpassing the uninoculated control (blank; pH 4.67). On the other hand, the solubilization of AlPO_4_ occurred without altering the pH, possibly as the initial pH of the culture was already low (4.6); this is a similar observation to that made in a previous evaluation of this phosphate [23].

### 4.3. Solubilization of FePO_4_ by Rhizospheric Bacteria

Iron phosphate (FePO_4_) is known to be one of the most challenging compounds to solubilize by rhizospheric bacteria. In our research, three strains stood out in terms of iron phosphate solubilization ability: Sfc-159-01, Sfcv-041-9, and *Enterobacter* sp. Sfcr-043-02 (PP319606), which achieved solubilization levels of 37.31 mg L^−1^, 34.96 mg L^−1^, and 32.61 mg L^−1^, respectively. Similar results were reported by [8] and [31], with maximum solubilization levels of 199.00 mg L^−1^ and 84.15 mg L^−1^, respectively. It is worth noting that our study constitutes a novel report on iron phosphate solubilization by native strains originating from extremely acidic soils, with a pH ranging between 3.99 and 4.62 [18]. 

Regarding the pH of the cultured supernatant, a negative correlation was observed between the soluble phosphorus and its pH (Table 3). Similar results were reported by [8] and [31], noting a decrease in the medium pH for all isolates evaluated compared to the uninoculated control.

The high solubilization potential of the bacteria identified in this study is attributed to their ability to produce and release substances that facilitate the conversion of insoluble phosphates into soluble forms. According to [14], this process can be carried out through various mechanisms such as the production of organic acids (citric acid, acetic acid, lactic acid, and gluconic acid) that lower the pH of the immediate environment, causing the dissolution of insoluble phosphate compounds such as iron and aluminum phosphates, converting them into soluble phosphate forms. Another process involves the release of enzymes such as phosphatases and phytases, which catalyze the release of phosphorus from organic and inorganic compounds, making it available for plant uptake [12,13]. These bacteria can also produce chelating compounds (siderophores) that bind to cations such as iron and aluminum. This reduces the formation of insoluble phosphates and releases phosphorus in soluble forms [14]. Additionally, phosphate-solubilizing bacteria can participate in other biochemical processes, such as the reduction of Fe(III) to Fe(II), which can also contribute to phosphorus solubilization [6].

Acidic soils are characterized by nutrient deficiencies, especially with respect to phosphorus [32]. Our results highlight the presence of rhizospheric bacteria with a high solubilization potential, which is attributed to the unique ability of legumes to establish two types of symbiosis: mycorrhizal and rhizobial. Rhizospheric bacteria associated with these plants could meet the demand for phosphorus, as this nutrient plays a crucial role in the symbiotic nitrogen fixation process, thus being essential for their growth and development [33,34]. Furthermore, legumes deploy different morphological and biochemical mechanisms to enhance phosphorus acquisition under acidic soil conditions, thereby increasing their capacity to fix atmospheric nitrogen [35].

### 4.4. Molecular Characterization of Rhizospheric Bacteria

The molecular analysis of the 16S ribosomal gene of the four isolates revealed that two of the strains belong to the two generic groups of *Enterobacter* sp. and *Pseudomonas* sp., while the other two correspond to *Staphylococcus saprophyticus*. It has been reported that *Enterobacter* sp. exhibit high potential for phosphate solubilization in other plant rhizospheres [13,36,37], although there is limited information about their solubilizing activity in association with cover legumes.

On the other hand, *Pseudomonas* sp. have been isolated and identified as some of the most efficient phosphate-solubilizing bacteria [13,38], as well as those from the genera *Staphylococcus*, *Micrococcus*, *Streptomyces*, and *Bacillus* [39]. Furthermore, of the four identified strains, three of them—*Pseudomonas* sp. Sfcv-041-01-2 (PP319610), *Staphylococcus saprophyticus* Sfc-093-04 (PP319607), and *Staphylococcus saprophyticus* Sfcv-098-02 (PP319608)—exhibited a high capacity for aluminum phosphate solubilization, while *Enterobacter* sp. Sfcr-043-02 (PP319606) demonstrated high iron phosphate solubilization ability.

## 5. Conclusions

The findings of this study have significant implications in the field of sustainable agriculture and soil fertility improvement in areas affected by acidic soils. The ability of rhizospheric bacteria isolated from cover legumes to solubilize inorganic phosphates such as aluminum phosphate and iron phosphate holds promise for addressing phosphorus deficiencies in acidic soils. The identification of specific bacterial strains, such as *Enterobacter* sp., *Pseudomonas* sp., and *Staphylococcus* sp., which demonstrated high efficacy in phosphate solubilization, provides valuable information for the development of microbial products and specific agricultural practices. Additionally, the observation of a decrease in pH during solubilization reactions suggests the release of organic acids by bacteria, further contributing to phosphate solubilization. This finding provides additional insights into the underlying mechanisms involved in phosphate solubilization by these bacteria and could lead to future research on optimizing conditions to enhance their efficacy.

Given their effectiveness, these strains could be utilized to address soil phosphorus deficiencies, thus promoting sustainable agricultural production in the Cumbaza River Sub-basin, San Martín region, Peru. Despite the promising findings of the study, there are some limitations that could influence the interpretation of the results and their applicability. Some of these potential limitations include the following. Sample size: the study may have been limited by the sample size or the selection of study areas. Bacterial diversity: although several phosphate-solubilizing bacterial strains were identified and characterized, there may be greater bacterial diversity in the rhizosphere that was not explored in this study. Effects of the culture medium: the study results may have been influenced by the type of culture medium used for isolating phosphate-solubilizing bacteria. Lack of long-term analysis: the study focused on the bacteria’s ability to solubilize phosphates over a specific period of time. Addressing these potential limitations in future research could provide a more comprehensive understanding of the efficacy and applicability of phosphate-solubilizing bacteria in improving phosphorus availability in acidic soils and thus promoting sustainable agricultural production.

## Figures and Tables

**Table 1:** Origins of rhizospheric bacteria strains isolated from cover legumes growing in acidic soils of the Cumbaza sub-basin.

Areas	*Vigna unguiculata*	*Canavalia ensiformis*	*Crotalaria juncea*
Chirikyacu	-	Sfcv-001-01	Sfcr-003-02
	-	Sfcv-017-03	-
Vista Alegre	Sfc-054-02	-	-
	Sfc-070-04	-	-
Chontal	Sfc-159-01	Sfcv-041-01-2	Sfcr-043-02
	-	Sfcv-041-9	-
San Antonio de Cumbaza	-	Sfcv-129-01	Sfcr-123-01
Shapumba	-	Sfcv-098-02	Sfcr-116-01
Aucaloma	Sfc-093-04	Sfcv-082-11	-
	-	Sfcv-089-01	-

**Table 2:** Morphocolonial characteristics of bacteria isolates obtained from the rhizosphere of cover legumes growing in acidic soils of the Cumbaza sub-basin.

Strains	Microscopic Features		Macroscopic Features						
	Gram Staining	Bacteria Shape	Colony Diameter (mm)	Color	Surface	Elevation	Colony Shape	Margin	Consistency
Sfc-054-02	−	bacillus	2.17	cream	smooth	P	Ci	En	creamy
Sfc-070-04	−	bacillus	1.04	cream	smooth	P	Ci	En	creamy
Sfc-159-01	−	bacillus	2.15	cream	smooth	P	Ci	En	creamy
Sfc-093-04	+	coccus	0.99	orange	smooth	P	Ci	En	creamy
Sfcv-001-01	−	bacillus	0.82	cream	smooth	C	Ci	En	creamy
Sfcv-017-03	−	bacillus	0.9	cream	smooth	P	Ci	En	creamy
Sfcv-041-01-2	−	bacillus	3.86	brown	smooth	P	Fu	L	creamy
Sfcv-041-9	−	bacillus	1.39	cream	smooth	P	Ci	En	creamy
Sfcv-129-01	−	bacillus	0.87	cream	smooth	P	Ci	En	creamy
Sfcv-098-02	+	coccus	1.08	orange-yellow	smooth	P	Ci	En	creamy
Sfcv-082-11	−	bacillus	1.48	cream	smooth	P	Ci	En	creamy
Sfcv-089-01	−	bacillus	1.67	cream	smooth	P	Ci	En	creamy
Sfcr-003-02	+	coccus	0.38	cream	smooth	P	Ci	En	creamy
Sfcr-043-02	−	bacillus	1.05	cream	smooth	P	Ci	En	creamy
Sfcr-123-01	−	bacillus	0.82	cream	smooth	P	Ci	En	creamy
Sfcr-116-01	−	bacillus	1.26	cream	smooth	P	Ci	En	creamy

Gram Staining: + = Gram-positive, − = Gram-negative; Colony Diameter: Diameter reached at 7 days of incubation; Elevation: P = Flat, C = Convex; Shape: Ci = Circular, Fu = Fusiform; Edge: En = Entire, L = Lobulated.

**Table 3:** Evaluation of AlPO_4_ and FePO_4_ solubilization by rhizospheric bacteria.

Strains	AlPO_4_		FePO_4_	
	pH	Solubilized P (mg L^−1^)	pH	Solubilized P (mg L^−1^)
CIAT 899	4.86 (±0.02) b	NPS	4.26 (±0.01) c	NPS
Sfc-054-02	4.18 (±0.08) efg	15.81 (±0.77) cde	4.08 (±0.05) cd	28.97 (±1.80) cd
Sfc-070-04	4.41 (±0.02) d	NPS	3.29 (±0.04) e	17.22 (±1.18) e
Sfc-093-04	5.23 (±0.14) a	26.50 (±1.58) b	4.02 (±0.01) cd	NPS
Sfc-159-01	4.34 (±0.02) de	17.09 (±2.37) cd	3.73 (±0.01) cde	37.31 (±0.90) a
Sfcv-001-01	4.81 (±0.02) bc	NPS	3.85 (±0.01) cde	25.98 (±0.33) d
Sfcv-017-03	4.08 (±0.07) fg	9.40 (±1.76) ef	3.86 (±0.00) cde	32.18 (±1.07) bc
Sfcv-041-01-2	5.21 (±0.00) a	55.98 (±6.27) a	5.37 (±0.02) b	15.36 (±1.86) e
Sfcv-041-9	4.03 (±0.06) g	6.41 (±0.61) fg	3.83 (±0.01) cde	34.96 (±1.32) ab
Sfcv-082-11	4.88 (±0.05) b	NPS	3.86 (±0.03) cde	29.19 (±1.47) cd
Sfcv-089-01	4.80 (±0.03) bc	NPS	3.95 (±0.04) cd	30.68 (±2.08) c
Sfcv-098-02	4.63 (±0.03) c	22.65 (±0.55) bc	3.89 (±0.02) cd	NPS
Sfcv-129-01	4.14 (±0.03) fg	5.98 (±1.47) fg	4.08 (±0.01) cd	NPS
Sfcr-003-02	4.37 (±0.11) d	12.61 (±0.92) def	4.24 (±0.67) c	26.50 (±1.52) d
Sfcr-043-02	4.25 (±0.08) def	NPS	3.54 (±0.01) de	32.61 (±0.51) bc
Sfcr-123-01	4.34 (±0.02) de	NPS	3.51 (±0.01) de	30.47 (±1.09) c
Sfcr-116-01	4.41 (±0.03) d	NPS	3.56 (±0.00) de	30.90 (±0.39) c
CV (%)	2.28	3.42	6.25	2.37

CIAT 899 = Positive control *Rhizobium tropici* CIAT 899. NPS = No phosphate solubilization. Duncan’s test (*p* < 0.01), means with different letters differ statistically from each other. CV = Coefficient of Variation.

**Table 4:** Percentage of similarity of the 16S rDNA gene of rhizospheric bacteria strains isolated from the rhizosphere of cover legumes.

Strains	Place of Origin	Host Legume	Solubilizing Phosphate	Most Related Species	Similarity (%)	Identified Strain/Accession Number in GenBank
Sfcv-041-01-2	Chontal	*Canavalia ensiformis*	AlPO_4_	*Pseudomonas aeruginosa* JCM 5962^T^/ *Pseudonomas paraeruginosa* PA7^T^	99.93	*Pseudomonas* sp. Sfcv-041-01-2/PP319610
Sfc-093-04	Aucaloma	*Vigna unguiculata*	AlPO_4_	*Staphylococcus saprophyticus* ATCC 15305^T^	100	*Staphylococcus* *saprophyticus* Sfc-093-04/PP319607
Sfcv-098-02	Shapumba	*Canavalia ensiformis*	AlPO_4_	*Staphylococcus saprophyticus* ATCC 15305^T^	100	*Staphylococcus* *saprophyticus* Sfcv-098-02/PP319608
Sfcr-043-02	Chontal	*Crotalaria juncea*	FePO_4_	*Enterobacter Sichuanensis* WCHECL1597^T^/*Enterobacter roggenkampii* DSM 16690^T^/ *Enterobacter quasiroggenkampii* WCHECL 1060^T^/*Enterobacter vonholyi* E13^T^	99.86	*Enterobacter* sp. Sfcr-043-02^T^/PP319606

## Data Availability

The molecular datasets generated during the current study are available in the NCBI GenBank, with accession numbers PP319610, PP319607, PP319608, and PP319606. Other datasets used during the present study are available from the corresponding author upon reasonable request.
